# Advances in AI-Driven EEG Analysis for Neurological and Oculomotor Disorders: A Systematic Review

**DOI:** 10.3390/bios16010015

**Published:** 2025-12-24

**Authors:** Faisal Mehmood, Sajid Ur Rehman, Asif Mehmood, Young-Jin Kim

**Affiliations:** 1 Department of AI and Software, Gachon University, Seongnam-si 13120, Republic of Korea; 2 Department of Creative Technologies, Air University, Islamabad 44000, Pakistan; 3 Department of Biomedical Engineering, Gachon University, Seongnam-si 13120, Republic of Korea; 4 Medical Device Development Center, Osong Medical Innovation Foundation, Cheongju 28160, Republic of Korea

**Keywords:** electroencephalography, deep learning, oculomotor analysis, neural signal processing, brain-computer interfaces, 68T07

## Abstract

Electroencephalography (EEG) has emerged as a powerful, non-invasive modality for investigating neurological and oculomotor disorders, particularly when combined with advances in artificial intelligence (AI). This systematic review examines recent progress in machine learning (ML) and deep learning (DL) techniques applied to EEG-based analysis for the diagnosis, classification, and monitoring of neurological conditions, including oculomotor-related disorders. Following the PRISMA guidelines, a structured literature search was conducted across major scientific databases, resulting in the inclusion of 15 peer-reviewed studies published over the last decade. The reviewed works encompass a range of neurological and ocular-related disorders and employ diverse AI models, from conventional ML algorithms to advanced DL architectures capable of learning complex spatiotemporal representations of neural signals. Key trends in feature extraction, signal representation, model design, and validation strategies are synthesized here to highlight methodological advancements and common challenges. While the reviewed studies demonstrate the growing potential of AI-enhanced EEG analysis for supporting clinical decision-making, limitations such as small sample sizes, heterogeneous datasets, and limited external validation remain prevalent. Addressing these challenges through standardized methodologies, larger multi-center datasets, and robust validation frameworks will be essential for translating EEG-driven AI approaches into reliable clinical applications. Overall, this review provides a comprehensive overview of current methodologies and future directions for AI-driven EEG analysis in neurological and oculomotor disorder assessment.

## 1. Introduction

Research on neurological and oculomotor disorders is undergoing a significant transformation through the integration of electroencephalography (EEG) with machine learning (ML) and deep learning (DL) techniques [1]. Advances in brain signal processing have enabled researchers to detect subtle alterations in neural activity associated with a wide range of neurological conditions, including those affecting eye movement control, thereby opening new avenues for early diagnosis and improved clinical management [2]. In recent years, increasingly sophisticated DL architectures have been applied to model the complex spatiotemporal characteristics of EEG signals across diverse neurological disorders, including oculomotor dysfunctions [3]. This shift represents a paradigm change in how neurological and eye-movement-related disorders are assessed, monitored, and managed [4].

EEG-based vision and oculomotor research is inherently interdisciplinary, lying at the intersection of medicine, neurophysiology, and computer science, and aims to elucidate the relationship between brain activity and eye movement behavior [5]. Traditionally, oculomotor and related neurological disorders have been diagnosed primarily through clinical examinations and behavioral observations, which can be subjective and may delay intervention [6]. With the emergence of AI-driven EEG analysis, it has become possible to identify subtle neural signatures that may precede overt clinical manifestations of neurological or oculomotor dysfunction [7]. The integration of artificial intelligence (AI) with neurophysiological data therefore holds substantial promise for enabling earlier diagnosis and more personalized therapeutic strategies across a broad spectrum of neurological disorders [8].

The availability of large-scale and benchmark datasets has further accelerated progress in this area. For instance, the introduction of the EEGEyeNet dataset provided standardized resources for investigating EEG–eye movement relationships and facilitated the development of data-driven models for neurological and oculomotor analysis [9]. Alongside dataset development, a variety of ML techniques have been proposed to improve diagnostic accuracy and prognostic assessment in neurological conditions [10]. While conventional ML methods such as support vector machines (SVMs) and random forest (RF) classifiers have demonstrated encouraging results, more advanced DL approaches—including convolutional neural networks (CNNs) and transformer-based models—have shown superior ability to capture complex nonlinear and spatiotemporal patterns inherent in EEG data [11]. These methodological advances have expanded the range of potential clinical applications and motivated comprehensive reviews of existing approaches and findings in the literature [12].

Despite these advances, significant challenges remain in applying EEG-based AI methods to neurological and oculomotor disorder assessment. Issues related to patient monitoring, data heterogeneity, and the scalability of real-time EEG analysis persist [13]. Moreover, translating algorithms developed in laboratory settings into clinical environments requires careful consideration of computational efficiency, robustness to population variability, and real-time processing constraints [14]. A thorough understanding of these limitations is essential to fully realize the potential of EEG-derived biomarkers for the evaluation and long-term monitoring of neurological and oculomotor disorders.

Recent studies have highlighted growing interest in high-precision neural analysis enabled by DL models, particularly those incorporating attention mechanisms and transformer architectures [15,16]. While these models often achieve high predictive performance, they also raise important concerns related to reliability, interpretability, data privacy, and clinical validation [17]. At the same time, advances in signal processing and adaptive modeling have enabled more responsive and continuous assessment frameworks, which are especially relevant for longitudinal monitoring of neurological conditions. As EEG-based AI methodologies continue to mature, the goal of delivering more individualized and precise neurological assessments becomes increasingly attainable.

The growing adoption of hybrid architectures that combine traditional ML techniques with modern DL frameworks reflects an important trend in EEG-based neurological research [18,19]. In particular, transformer-based attention models have emerged as powerful tools for modeling long-range dependencies in EEG signals, offering new possibilities for improving diagnostic accuracy and treatment personalization. These developments represent the state of the art in AI-driven EEG analysis for neurological disorders, including those involving oculomotor control. EEG-based machine learning studies explicitly targeting classical oculomotor disorders such as congenital nystagmus or strabismus remain limited in the current literature. Accordingly, this review focuses on neurological and oculomotor conditions in which eye-movement dysfunction is explicitly analyzed or modeled using EEG signals.

Accordingly, this systematic review aims to examine recent ML and DL approaches applied to EEG-based analysis of neurological and oculomotor disorders. The review focuses on contemporary model architectures, feature extraction strategies, and evaluation practices, with particular emphasis on the integration of advanced DL models with traditional ML paradigms. By systematically analyzing the existing literature, this study seeks to identify prevailing methodological trends, assess reported diagnostic performance, and highlight open challenges that warrant further investigation.

The primary contributions of this systematic review are as follows:

- Compilation of frequently adopted ML and DL model architectures applied to EEG data for neurological and oculomotor disorder assessment.
- Comparative analysis of reported performance metrics across different neurological and oculomotor conditions.
- Review of feature extraction, signal representation, and classification strategies employed in prior studies.
- Assessment of clinical relevance and reliability with respect to generalizability, computational efficiency, and real-time applicability.
- Identification of dataset limitations and methodological gaps that may be addressed through emerging DL and hybrid modeling approaches.
- Discussion of future research directions for EEG-based neurological and oculomotor disorder analysis using AI-driven frameworks.

The timing of this review is particularly appropriate given the recent advances in transformer-based models and attention mechanisms applied to brain signal analysis. Understanding how these developments can support more accurate diagnosis, real-time monitoring, and personalized intervention strategies is essential for advancing EEG-based AI applications in neurological and oculomotor healthcare.

## 2. Materials and Methods

This review was conducted in accordance with the Preferred Reporting Items for Systematic Reviews and Meta-Analyses (PRISMA) guidelines to ensure transparency, reproducibility, and methodological rigor. The objective was to systematically identify, screen, and synthesize existing studies that apply machine learning (ML) and deep learning (DL) techniques to electroencephalogram (EEG) data for the analysis and diagnosis of eye movement disorders (EMDs). Given the heterogeneity of datasets, models, and evaluation metrics across studies, a qualitative systematic review with structured narrative synthesis was performed, rather than a statistical meta-analysis. We included studies that applied EEG-based ML and DL methods to both neurological disorders and oculomotor-related conditions, provided that EEG constituted a primary modality for analysis.

### 2.1. Search Strategy

A comprehensive literature search was carried out across four major scientific databases: PubMed, IEEE Xplore, arXiv, and Google Scholar. These databases were selected to capture peer-reviewed biomedical literature as well as recent advances in engineering and computational intelligence. The search was conducted for studies published between 2015 and 2025 and restricted to articles written in English.

The search strategy combined controlled vocabulary terms (where applicable) with free-text keywords related to EEG, eye movement disorders, and artificial intelligence methodologies. Boolean operators were used to structure the queries.

#### Database-Specific Search Query

The following reproducible Boolean query was applied to the PubMed database:

(“Strabismus” OR “Nystagmus” OR “Amblyopia” OR “Oculomotor disorder” OR “Eye movement disorder” OR “Neuro-ophthalmology” OR “Ocular motility” OR “Eye tracking” OR “Visual dysfunction”) AND (“EEG” OR “Electroencephalogram” OR “Electroencephalography” OR “Brain signals” OR “Neural signals”) AND (“Machine learning” OR “Deep learning” OR “Artificial intelligence” OR “Neural networks” OR “Pattern recognition”) AND (“Diagnosis” OR “Detection” OR “Classification” OR “Assessment”)

Equivalent keyword combinations adapted to the syntax and indexing mechanisms of IEEE Xplore, arXiv, and Google Scholar were used. The complete set of retrieved records from all databases formed the initial pool for screening.

### 2.2. Study Selection Process

The study selection followed a multi-stage screening procedure consistent with the PRISMA guidelines. First, duplicate records were removed. Second, titles and abstracts were screened to exclude clearly irrelevant studies. Third, full-text articles were assessed for eligibility based on predefined inclusion and exclusion criteria.

**Figure 1:**
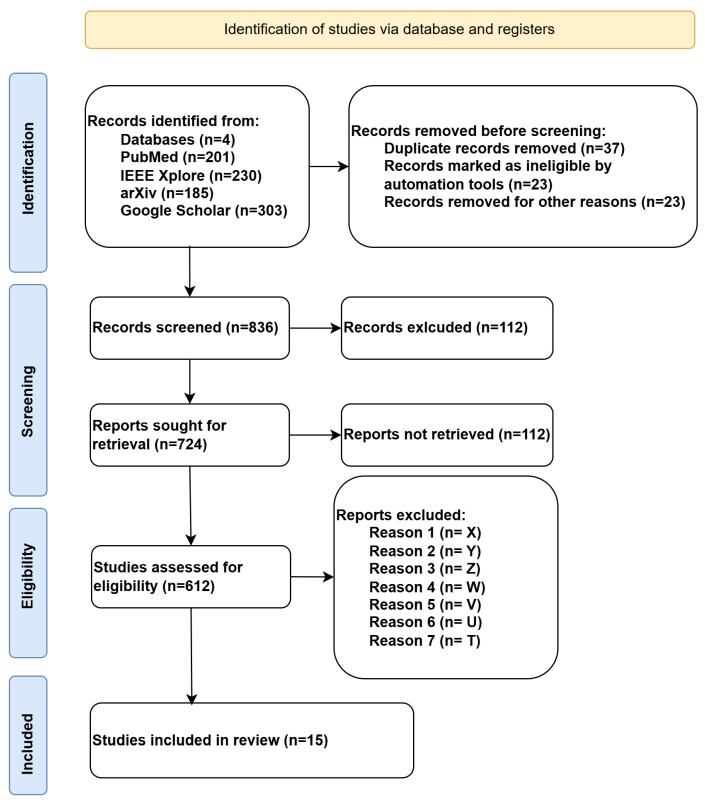
PRISMA flow diagram illustrating the screening and inclusion criteria.

Two authors independently performed the screening process. Any disagreements were resolved through discussion, and a third reviewer was consulted when consensus could not be reached. The detailed article selection flow, including reasons for exclusion at each stage, is illustrated in the PRISMA flow diagram in Figure 1.

### 2.3. Inclusion Criteria

Studies were included if they satisfied all of the following conditions:

- Peer-reviewed journal or conference articles.
- Use of EEG data as a primary or core modality.
- Application of ML and/or DL algorithms for analysis, classification, or diagnosis.
- Explicit focus on eye movement disorders, oculomotor dysfunctions, or closely related visual–neurological conditions.
- Clear description of model architecture, feature extraction methods, and evaluation metrics.

Studies focusing on neurological disorders without explicit oculomotor endpoints were included when they contributed methodological insights relevant to EEG-based disorder classification and neural signal interpretation.

For the purpose of this review, oculomotor disorders and oculomotor-related tasks are defined as conditions or experimental paradigms in which abnormalities of eye movements—such as saccades, fixation, gaze control, blinks, or visual tracking—constitute a primary clinical endpoint or a directly analyzed neural function. Broader neurological or psychiatric disorders were included only when eye-movement dysfunction was explicitly analyzed using EEG, or when EEG-based tasks probed neural mechanisms related to oculomotor control, rather than incidental or secondary ocular involvement, with or without auxiliary eye-tracking validation.

### 2.4. Exclusion Criteria

Studies were excluded if they met any of the following criteria:

- Did not involve EEG data or did not apply ML/DL techniques.
- Focused solely on eye-tracking or imaging modalities without EEG integration.
- Lacked sufficient methodological details or quantitative performance evaluation.
- Were non-peer-reviewed articles, editorials, reviews, or opinion papers.
- Were not written in English or were published outside the defined time window.

### 2.5. Data Extraction and Synthesis

From each included study, structured data were extracted, including publication year, dataset characteristics, sample size, EEG acquisition details, feature extraction techniques, ML/DL models employed, performance metrics, and reported clinical relevance. A pilot extraction was conducted on a random subset of five studies to ensure consistency and reliability.

Given the methodological diversity and variability in outcome measures, a formal meta-analysis was not feasible. Instead, a qualitative synthesis was performed, grouping studies according to their ML/DL architectures, feature extraction strategies, and targeted EMD categories. Comparative analysis focused on trends in model performance, architectural evolution from conventional ML to DL approaches, and reported clinical applicability.

### 2.6. Bias Assessment

Risk of bias was qualitatively assessed by examining dataset size, validation strategy (e.g., cross-validation vs. external validation), and transparency of reporting. Studies relying on small datasets or lacking independent validation were noted as potential sources of bias. This assessment informed the interpretation of our findings, rather than serving as exclusion criteria.

### 2.7. Reporting and Visualization

The characteristics and outcomes of the included studies are summarized in tabular form to facilitate comparison. Descriptive statistics related to publication trends, database distributions, and thematic categorization are provided to contextualize the research landscape. These summaries are intended to support narrative interpretation, rather than to imply quantitative aggregation of results.

The PRISMA flow diagram summarizing the identification, screening, eligibility, and inclusion stages is presented in Figure 1.

## 3. Results

The results are presented at two complementary analytical levels: First, corpus-level analyses are used to contextualize research trends, publication patterns, and methodological emphasis observed across the broader screened literature (*n*= 836). Second, study-level analyses focus exclusively on the final 15 included studies that met the PRISMA eligibility criteria, providing detailed synthesis of datasets, models, features, and clinical applications. All figures and tables are explicitly interpreted according to this distinction so as to avoid ambiguity between contextual trends and evidence derived from the included studies.

To further clarify the scope of the included literature, the final 15 studies were heterogeneous in their relationship to oculomotor function and can be conceptually grouped into three categories: (i) studies directly analyzing eye movement or oculomotor control using EEG, including gaze, fixation, blinks, eye-state recognition, and EEG–eye-tracking integration; (ii) neurological disorder studies, in which oculomotor dysfunction was a recognized clinical manifestation and EEG was used to analyze related neural correlates; and (iii) a limited number of eye-related studies included primarily for their methodological contributions to EEG-based or AI-driven disease classification rather than direct investigation of oculomotor pathology. This distinction is provided to ensure transparency regarding study inclusion and to contextualize the interpretation of figures and study-level results presented in the remainder of this section.

The systematic review flowchart in Figure 1 outlines the process of identifying, screening, and including studies related to EEG-based EMDs using ML and DL. The study selection process began with the identification of 836 records from four databases: PubMed (201), IEEE Xplore (230), arXiv (185), and Google Scholar (303). Before screening, 83 records were removed due to duplication, ineligibility detected by automation tools, or other reasons. The exclusion criteria are presented in Figure 1.

Reason 1 (*n* = X): Irrelevant population (e.g., animal studies, non-human EEG).
Reason 2 (*n* = Y): Ineligible study design (e.g., reviews, editorials, non-comparative studies).
Reason 3 (*n* = Z): No full text available.
Reason 4 (*n* = W): Ineligible intervention/comparison (e.g., no ML applied to EEG data).
Reason 5 (*n* = V): Outcome not measured/reported (e.g., no diagnostic performance metrics).
Reason 6 (*n* = U): Duplicates (beyond initial screening).
Reason 7 (*n* = T): Language barrier (if applicable and stated in the methodology).

During the subsequent screening process, the researchers evaluated 836 records and eliminated 112 that did not satisfy the study requirements or appeared irrelevant. Out of all reports, 724 faced retrieval attempts, but 112 remained unretrieved because of reasons including access restrictions or missing full texts. The eligibility assessment of 724 reports led to multiple exclusions because of factors X, Y, Z, and W, which narrowed down the qualifying studies to only 15. Through its intricate methodology, this structured process applies rigorous selection criteria to eliminate irrelevant, low-quality studies while ensuring that only the most pertinent research is included for an exhaustive literature review on EEG-based EMD analysis using AI techniques. Scientific works clustered between 2015 and 2025 demonstrate a recent escalation of interest in EMD research.

**Figure 2:**
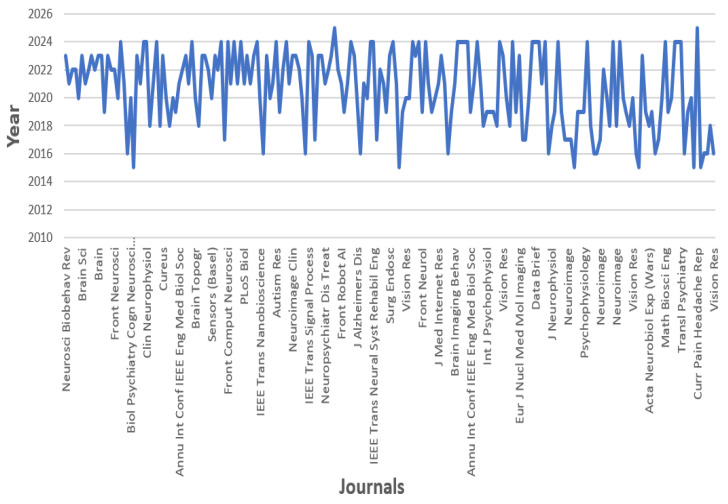
Corpus-level publication trends from the PubMed database related to EEG-based neurological and eye movement disorder research over the past 10 years.

Figure 2 signifies the publication trend analysis of EEG-based EMD research using ML and DL in peer-reviewed journal articles. The *x*-axis contains a list of journals that published studies related to EMDs, while the *y*-axis indicates publication years and ranges from 2015–2025. The changing line shows the number of published papers over time, indicating patches of intense or low activity in these journals.

**Figure 3:**
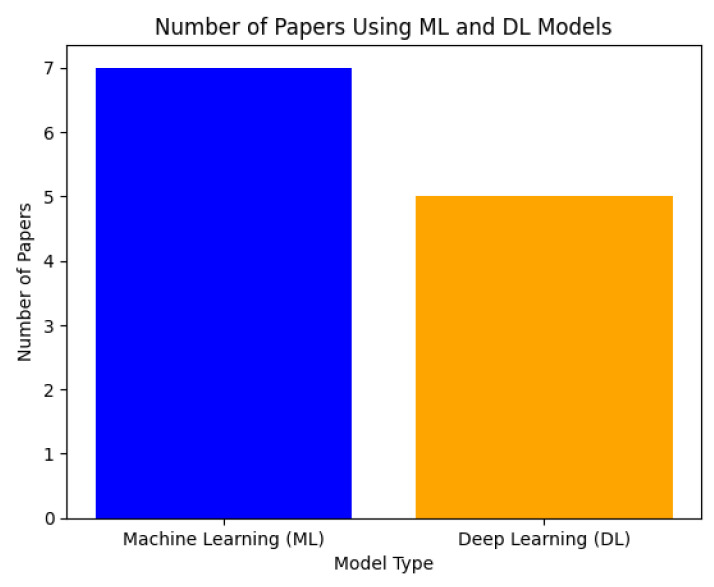
Overall distribution of machine learning and deep learning models reported in the EEG-based neurological and eye movement disorder literature.

Figure 3 shows how many research papers incorporate ML and DL models for EMDs. The data demonstrates that researchers published more papers using ML-based methods than with DL-based methods. ML techniques were employed in EEG-based predictive studies regarding pheno-conversion in sleep disorders, as well as alcohol use disorder (AUD) detection and MDD diagnosis. DL techniques became more prominent for classification tasks, including EEG data segmentation and eye-state recognition, as well as the detection of retinal diseases from fundus images. Research papers that combined both ML and DL techniques contributed to their designated category totals. ML models achieved broader acceptance for their interpretability and diverse dataset applicability, whereas DL methods showed a preference for image-based classification tasks.

**Figure 4:**
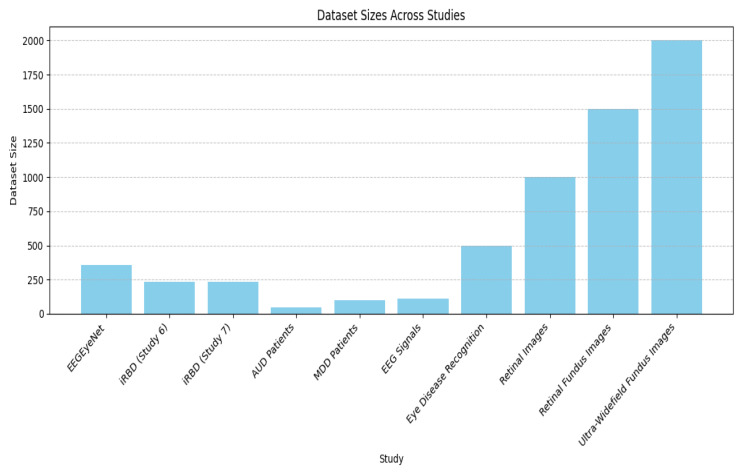
Distribution of dataset sizes reported in the 15 studies included in the systematic review.

As shown in Figure 4, dataset sizes vary significantly across studies. Studies related to retinal images and fundus images have the largest datasets, with ultra-wide-field fundus images reaching 2000 samples, followed by retinal fundus images (RFIs) with 1500 samples and retinal images with 1000 samples. EEG-based studies tend to have smaller datasets, as EEGEyeNet has the largest dataset among EEG-related studies, with about 350 samples, whereas other EEG-related datasets—such as those for idiopathic REM Sleep Behavior Disorder (iRBD) in Studies 6 and 7, Major Depressive Disorder (MDD) patients, and EEG signals—contain fewer than 300 samples each. Each study’s data distribution indicates that ophthalmology research gains from extensive datasets thanks to large-scale image availability, while EEG studies depend on smaller samples due to neurological data collection difficulties.

**Figure 5:**
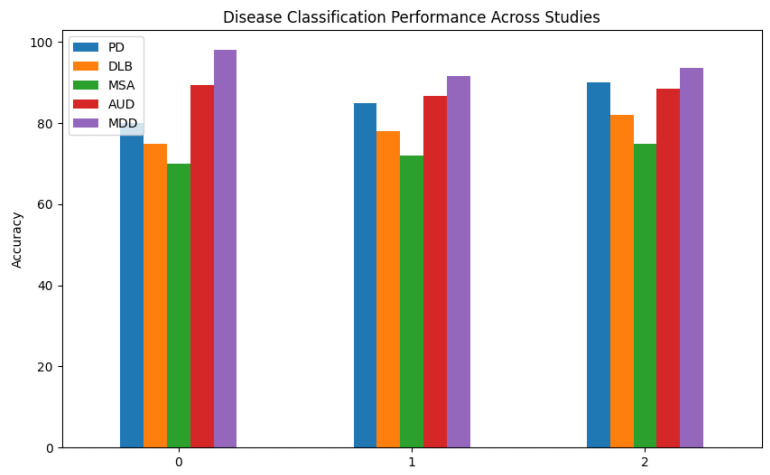
Distribution of diseases reported in EEG-based neurological and oculomotor studies.

In Figure 5, the classification performance of different disease categories from the 15 selected papers is shown. The diseases targeted in these studies include Parkinson’s disease (PD), Dementia with Lewy Bodies (DLB), Multiple System Atrophy (MSA), AUD, and MDD. The studies focus on EEG-based models for disease classification, given the relevance of EEG in neurological and psychiatric disorders.

- EEG is frequently used to diagnose Parkinson’s disease (PD), a progressive neurodegenerative disorder that affects mobility, by detecting abnormal brain activity associated with motor function and cognitive decline [20].
- Dementia with Lewy Bodies (DLB) is closely related to PD, although it has different cognitive and behavioral symptoms; EEG can help distinguish DLB from other dementias [21].
- EEG analysis helps differentiate MSA from PD and DLB. MSA is a rare neurological disorder that is comparable to Parkinson’s but involves broad autonomic and movement dysfunction [22].
- EEG studies frequently look at altered brain wave patterns in people with AUD, a chronic illness marked by an inability to control alcohol use [23].
- MDD is a severe mental health disorder that impacts mood, cognition, and daily functioning; EEG is widely used to study abnormal brain activity in MDD patients [24].

Our results from each of these investigations showed that the MDD had the best classification accuracy, coming in close to 100%, suggesting that EEG-based models could be useful for detecting brain activity associated with depression. Additionally, AUD is highly accurate, indicating that EEG accurately captures the neurophysiological changes caused by alcohol. PS’s EEG patterns are easier to identify than those of DLB, MSA, or PD, which has the highest diagnosis accuracy of the three. Because MSA’s EEG has the lowest accuracy and has characteristics that are similar to those of other disorders, classification becomes more challenging.

**Figure 6:**
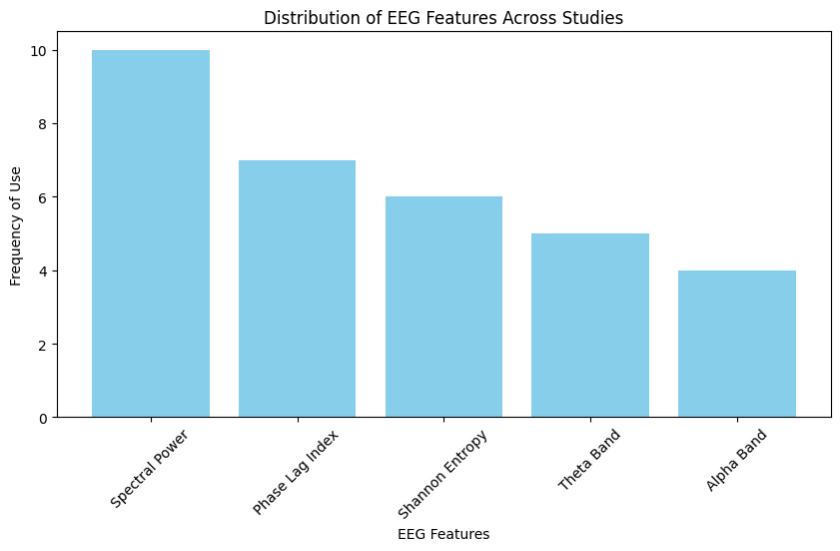
Distribution of EEG features across all studies, with the frequency of use of different EEG metrics used for the disease classification.

The most frequently used features in EEG-based classification of diseases are highlighted by the distribution of EEG features used in all 15 research studies. Since spectral power (SP) is the most prevalent component among the returned EEG data, it is crucial for examining the patterns of brain activity displayed in Figure 6. Other common features of the EEG signals are the phase lag index (PLI) and Shannon entropy (SE), which demonstrate their importance for expressing the intricacy and interrelationships of the signals of the EEG. Theta (Θ) and alpha (α) bands are also essential EEG-extracted features in these papers, due to their offering important insights into brain functions. The most important feature among all others is the power spectrum analysis, which is indicated by variance in feature utilization in EEG-based disease detection; entropy-based measures are also receiving greater focus due to their capacity to characterize channel complexities.

**Figure 7:**
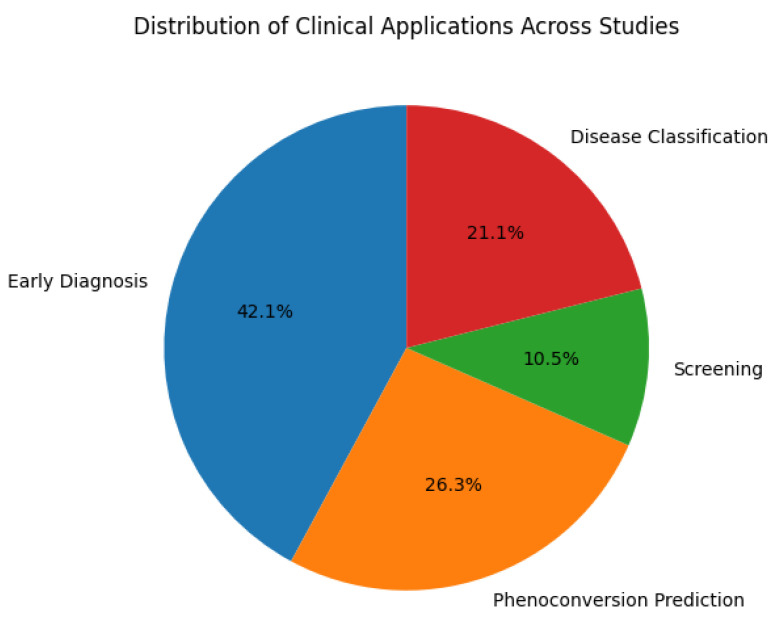
Spectrum of clinical uses demonstrating the main uses for EEG in assessment, illness categorization, and pheno-conversion forecasting, including early detection.

The pie chart in Figure 7 shows the distribution of clinical applications of the studies from Table 1, Table 2, Table 3, Table 4, Table 5, Table 6, Table 7, Table 8, Table 9, Table 10, Table 11, Table 12, Table 13, Table 14 and Table 15 of the EEG-based research that is utilized in different medical contexts. The largest portion, early diagnosis, is 42.1% in these studies and emphasizes the significant role of EEG in detecting diseases at an early stage, helping in timely interventions, and improving patient outcomes. A pheno-conversion forecast of 26.3% is a significant percentage that shows how well EEG predicts when a preliminary stage will give way to a full-blown illness, especially in neurodegenerative diseases. A second significant use that shows how EEG is used to differentiate among various neurological disorders is disease categorization (21.1%). The practicality of scanning in early evaluations to identify people at risk before a final diagnosis is highlighted by the fact that it has the lowest proportion, at 10%. The successful handling of neurological and mental illnesses depends on early identification and forecasting, and this is strongly emphasized in the process of distribution.

From the findings of the analysis of sex differences in mental rotation tasks, significant neural processing distinctions emerged between men and women. Women showed higher right parietal activation of 665.74 ± 444.01 μV, compared to men at 317.65 ± 412.59 μV, while behavioral measures revealed no sex differences in either reaction time or accuracy. The ET findings revealed that the different visual processing used by women is a fragmentary technique with more obsessions, while males used an integrated approach with overall longer static periods. The findings imply that men and women use essentially distinct intellectual and neurological pathways during mental shift responsibilities, even when their functional score is uniform.

Ref. [25] investigated the classification of resting EEG data in individuals with idiopathic Rapid EM Behavior Disorder (RBD) using DL approaches, specifically CNNs and RNNs (LSTM/GRUs). To determine whether RBD would progress to PD or Dementia with Lewy Bodies (DLB), the study detected two important EEG features: Θ band and decreased α band bursting. With the minimal preprocessing specified in Table 1, the model obtained a good classification accuracy of 80% and an AUC of 87%. DeepDream’s synthetic spectrograms provide additional information on crucial time–frequency characteristics for early diagnosis.

**Table 1:** Study 1: DL with EEG spectrograms in Rapid EM Behavior Disorder (RBD).

Category	Description
EEG Features	Resting EEG from RBD patients and controls. Focus on Θ and α band changes in RBD converters (PD or DLB).
Analysis Method	CNN and RNN models; 80% ± 1% accuracy; 87% ± 1% Area under the Curve (AUC) using the best EEG channel.
Clinical Application	Predicts RBD conversion to PD or DLB years in advance. Identifies biomarkers for α-synucleinopathies.

Using EEG and eye-tracking (ET) signals, researchers [26] developed a hybrid EEG–eye-tracker framework designed to automatically identify and remove eye movement and blink artifacts from electroencephalographic recordings. Table 2 summarizes the model design, analytical pipeline, and key results. The study demonstrates that integrating synchronized eye-tracking data with EEG enables highly accurate detection of ocular artifacts, outperforming traditional blind source separation and regression-based methods. This hybrid approach allows for improved preservation of neural signal integrity and paves the way for real-time, artifact-free EEG applications in both clinical and cognitive neuroscience contexts.

**Table 2:** Study 2: hybrid EEG–eye-tracking framework for automated artifact detection and removal.

Category	Description
ML/EEG Features	EEG signals recorded from 32–64 scalp electrodes combined with synchronized eye-tracking data capturing saccades, blinks, and gaze position. Eye-tracking events served as reference markers for identifying ocular components within EEG data.
Analysis Method	Automatic artifact detection using Independent Component Analysis (ICA) guided by eye-tracking-based spatial and temporal correlation analysis. Identified components corresponding to eye movements or blinks were removed, and a cleaned EEG was reconstructed. The hybrid approach improved artifact detection accuracy compared to ICA alone.
Performance/Outcomes	The hybrid EEG–ET model achieved near-perfect identification of ocular artifacts (≈98–99% detection accuracy) while minimizing distortion of cortical signals. It demonstrated superior performance in preserving event-related potentials relative to conventional regression or ICA-only techniques.
Clinical/Research Application	Provides a reliable method for real-time or offline artifact correction in EEG studies involving active eye movements, supporting cleaner neural analyses in cognitive, clinical, and neuroergonomic research.

A study that used R-S EEG data leveraged from machine learning models [27] is shown in Table 3, which predicted pheno-conversion time and subtype in patients with iRBD. Important characteristics, such as SP, PLI, and SE, show that EEG slowing is essential for subtype categorization and survival prediction from Table 3. Strong performance was demonstrated by the RSF and KNN models, with concordance indices and AUC values indicating good accuracy. Although bigger datasets are required for improved generalizability, external validation validates the possibility of early identification of neurodegenerative disorders.

**Table 3:** Study 3: pheno-conversion prediction using EEG-based machine learning models in idiopathic RBD.

Category	Description
ML/EEG Features	Resting EEG from iRBD patients. SP, weighted PLI, and SE. Key finding: EEG slowing is important for survival prediction and subtype classification models.
Analysis Method	Best model = RSF with Brier score of 0.114, concordance index = 0.775, KNN for subtype prediction with AUC of 0.901.
Clinical Application	Predicts when the subtype will change from iRBD to MSA, DLB, and PD and determines which patients most likely have the illness.

By integrating EEG and ET data from 356 subjects in three different experimental paradigms, the EEGEyeNet dataset was created to further research on brain activity and EMs [28]. This dataset aims to enhance the EM prediction provided in the study by integrating benchmark tasks such as left–right, angle–amplitude, and absolute position, as shown in Table 4. The performance of several big NNs and traditional ML models was assessed. The code and dataset’s user-friendly interface and public availability make them an invaluable resource for further EM research.

**Table 4:** Study 4: EEGEyeNet in studies on EMs and brain activity.

Category	Description
Dataset Name	EEGEyeNet
Goal	Advancing research in brain activities and EMs
Modalities	EEG (electroencephalography) and ET (eye-tracking)
Subjects	356
Experimental Paradigms	3 (pro-antisaccade, large grid, VSS)
Benchmark Tasks	3 (left–right, angle–amplitude, absolute position)
Models Evaluated	Classical ML and large NNs
Code and Data	Released with an easy-to-use interface

A DL framework called DETRtime was developed for time-series segmentation of EEG data in [29] to detect eye movements involving fixation, blinks, and leaps despite the need for ET data. State-of-the-art performance in ocular event detection was achieved by the model by merging computer vision techniques with an end-to-end DL scheme, which demonstrates outstanding generalization abilities and performs well when it comes to EEG sleep stage segmentation. Table 5 shows time-series data segments during visual detection of events from EEG with DETRtime.

**Table 5:** Study 5: time-series data segments during visual detection of events from EEG with DETRtime.

Category	Description
Framework Name	DETRtime
Goal	Ocular event detection using EEG
Modalities	EEG (electroencephalography)
Key Feature	Detects ocular events without requiring ET data
Segmentation Targets	Saccades, fixations, blinks
Methodology	End-to-end DL with computer vision techniques
Performance	Achieves state-of-the-art results in ocular event detection
Generalization	Effective in EEG sleep stage segmentation

Another research study [27] attempted to predict the pheno-conversion time and subtype of iRBD patients using baseline EEG features, as shown in Table 6. The cohort, which included 236 iRBD patients who were tracked for an average of 3.5 years, provided the EEG parameters SP, weighted PLI, and SE for analysis. The RSF model was the best for predicting survival, whereas KNN was the best at type predictions. EEG slowing was identified as a crucial element in the models’ external evaluation. The study highlights the potential of EEG patterns for predicting the course of sickness while also underscoring the need for more comprehensive, worldwide research.

**Table 6:** Study 6: employing preliminary EEG parameters to predict pheno-conversion interval and subtype in iRBD patients.

Category	Description
Study Objective	Use foundation EEG features for predicting pheno-conversion interval and subgroup in patients with iRBD.
Patient Group	Data of 236 iRBD patients for 8 years, with an average of 3.5 years.
Features Extracted from EEG	SP, weighted PLI, SE.
Prediction Models	Three models used for survival prediction and four for subtype.
Best Survival Prediction Model	RSF model Brier score = 0.114, and concordance index = 0.775.
Best Subtype Prediction Model	K-nearest neighbor (KNN) model with AUC = 0.901.
Important EEG Feature	Slowing of the EEG.
Validation	External validation using data from a different institution.
Conclusions	Baseline EEG features predict pheno-conversion time and subtype of patients.
Future Research	Larger studies with international datasets needed for robust models.

Using baseline EEG characteristics, this research used different ML models to predict the pheno-conversion time and subtype of α-synucleinopathy PD, MSA, and DLB in iRBD patients [27]. The dataset contained 233 patients with iRBD, with an average monitoring time of 4.1 years. The EEG characteristics analyzed were weighted PLI, SE, and SP. The KNN model remained the best at classifying subtypes, whereas the RSF model was the best at predicting survival from the dataset. The study included external validation performed on the models, and EEG slowing was a significant predictive characteristic. Table 7 shows that EEG biomarkers were used to predict the course of neurodegenerative diseases early on, but more research using bigger, international datasets is required.

**Table 7:** Study 7: foundation EEG-based forecasting of α-synucleinopathy pheno-conversion frequency and duration in iRBD individuals.

Category	Description
Study Objective	Leveraging baseline EEG data from iRBD individuals, developing a forecasting framework for α-synucleinopathy pheno-conversion with time and classification.
Patient Group	A total of 233 people suffering from iRBD who were monitored for up to 9 years, with an average of 4.1 years.
EEG Features	SP, weighted PLI, SE.
Prediction Models	Four approaches for subgroup predictions with PD-MSA, DLB, and three distinct models for forecasting survival.
Best Survival Model	RSF model, with a Brier score of 0.113 and a concordance index of 0.721.
Best Classification Model	KNN model with AUC of 0.908.
Important EEG Feature	EEG slowing.
Validation	Concordance index, Brier score, and AUC.
Conclusions	For validation, more extensive research with different kinds of foreign datasets is required.
Future Research	Larger studies with diverse big data in corroboration of the same domain.

The authors of [30], trained an ML method for detecting AUD patients using resting-state (RS-EEG) attributes. During each EO and EC condition, EEG data were collected that comprised 30 AUD individuals and 15 age-matched HCs. Attributes were extracted using inter-hemispheric coherence, and the δ, θ, α, β, and γ SP in various EEG channels were examined. The most optimal model had remarkable classification performance, with 89.3% accuracy, 88.5% sensitivity, and 91% specificity. The results of the EEG data, particularly in the Θ, β, Γ, and inter-hemispheric coherence bands, enable the study’s automated AUD screening, as shown in Table 8.

**Table 8:** Study 8: ML approach for automating EEG-based AUD patient detection.

Category	Description
Study Objective	Implement an ML technique that uses R-S EEG-derived properties to automatically evaluate AUD patients.
Patient Group	Fifteen age-matched normal controls and thirty patients with AUD.
EEG Recording Conditions	Five minutes of eye closed (EC) and five minutes of eye open (EO).
EEG Features Extracted	Inter-hemispheric coherences and SP in δ, Θ, α, β, Γ bands with 19 scalp locations.
Feature Selection Method	Leveraging receiver operating characteristic curves for rank-based selection of features.
Best Classification Results	Integration of EEG features Θ, β, Γ power, and inter-hemispheric coherence.
Classification Performance of Best Model	With accuracy = 89.3%, sensitivity = 88.5%, specificity = 91%, and F1-score = 0.90.
Alternative Results	EEG band power classification with accuracy = 86.6%, sensitivity = 95%, specificity = 82.5%, and F1-score = 0.88.
Conclusion	EEG data with the channel features Θ, β, Γ power, and inter-hemispheric coherence are objective markers for screening AUD patients.

Ref. [31] built a combination of ML models that recognize MDD employing EEG-derived SL attributes. The patient category participants in the study consisted of MDD patients and HCs. Table 9 shows several approaches to classification, including LR, SVM, and NB. Among all models, SVM had the best performance, with 98% accuracy, 99.9% sensitivity, and an F1-score of 0.97. The results indicate that SL has potential as a feature for medical evaluation integration and in automated MDD detection.

**Table 9:** Study 9: ML method for EEG synchronization-probability-based MDD detection.

Category	Description
Study Objective	Develop an ML model for the automatic recognition of MDD employing synchronization probability parameters extracted from EEG.
Patient Group	MDD patients and HCs.
EEG Feature Extracted	SL.
Classification Models	SVM, Logistic Regression (LR), Naïve Bayesian (NB).
Best Classification model: SVM	Accuracy = 98%, sensitivity = 99.9%, specificity = 95%, and F1-score = 0.97.
Classification Results of LR	Accuracy = 91.7%, sensitivity = 86.66%, specificity = 96.6%, and F1-score = 0.90.
Classification Results of NB	Accuracy = 93.6%, sensitivity = 100%, specificity = 87.9%, and F1-score = 0.95.
Conclusion	A potential method for identifying depressive disorders, SL can help develop practical diagnostic tools.

Ref. [32] used sophisticated EEGNet, deep ConvNet, and shallow ConvNet to identify ocular states (EO and EC) utilizing simultaneously collected ear-EEG and scalp-EEG data. With a detection duration of 2.35 s, a minimal FP rate of 0.29 FPs/min, and a TP rate of 93%, the classification job produced remarkable results. In order to improve accuracy for both clinical and practical applications, Table 10 shows how CNN models and Ear-EEG data can be used for real-time eye-state identification applications.

**Table 10:** Study 10: eye-state recognition using deep CNNs employing ear-EEG and scalp-EEG data.

Category	Description
ML/EEG Features	EEG Dataset = ear-EEG and scalp-EEG simultaneously recorded during EO and EC states. Data Analysis = EEGNet, ConvNet, and shallow ConvNet. EEG Features = power density and other frequency variations.
Analysis Method	DL Models = ensemble models of EEGNet, deep ConvNet, and shallow ConvNet. Task = eye-state, EO, and EC classification. Metrics = true positive (TP) rate 93%, FP rate 0.29 FPs/min, detection speed 2.35 s, and transfer rate 21.86 bits per minute.
Clinical Application	Early Diagnosis/Prognosis = real-life applications for eye-state identification using ear-EEG. Clinical Insights = improved classification accuracy for ear-EEG with CNN models.

Another study [33] provides an automated method for differentiating eye states (EO vs. EC) using EEG data from 109 individuals by combining machine learning techniques with frequency plots and recurring quantification assessments. The key features that were extracted from 64 EEG channels were recurrence rate, determination, chaos, laminarity, trapping time, and the longest horizontal lines. The study employed a variety of machine learning models, including LR, SVM, and RF. As shown in Table 11, LR had the best performance, with 97.27% accuracy and a 97.17% F1 score. This method is useful for automatic eye-state classification in real-world EEG applications.

**Table 11:** Study 11: eye-states classification through recurrence quantification evaluation and EEG dataset.

Category	Description
ML/EEG Features	EEG Dataset = PhysioNet database EEG signals of 109 subjects. Data Analysis = nonlinear analysis with recurrence plots and quantification. EEG Features = rate, determinism, entropy, laminarity, trapping time, and longest vertical line from 64 EEG channels.
Analysis Method	Models = LR, SVM, RF, KNN, Gaussian Naïve Bayes (Gnb), and adaptive boosting. Task = EO vs. EC classification. Metrics = LR: accuracy = 97.27%, F1-score = 97.17%, precision = 98.26%, recall = 96.36%, specificity = 98.18%.
Clinical Application	Early Diagnosis/Prognosis = automated eye-state classification for practical applications. Clinical Insights = developing EEG-based applications for eye state using LR identification.

In this work, eye disorders were identified using a DL-based model that uses an upgraded D-S evidence theory (ID-SET) to utilize TL and decision fusion [34]. The model showed impressive performance, with 92.37% accuracy, 0.878 Kappa, 0.914 F1 score, 0.945 precision, 0.89 recall, and 0.987 AUC. By eliminating biases and paradoxes, ID-SET enhances the model’s decision-making capabilities. By offering a potential tool for the early diagnosis and classification of a range of eye diseases, this research enhances the precision and reliability of clinical diagnoses, as shown in Table 12.

**Table 12:** Study 12: DL with Transfer Learning (TL) and D-S evidence theory for the detection of eye diseases.

Category	Description
ML/EEG Features	EEG Dataset = EEG, focuses on eye disease recognition. Data Analysis = Deep-NN with TL and decision fusion using D-S evidence theory. Features = ocular disease recognition by using improved D-S theory fusion.
Analysis Method	DL Models = TL and decision fusion ID-SET. Task = eye disease recognition and classification. Performance Metrics = accuracy = 92.37%, Kappa = 0.878, F1-score = 0.914, precision = 0.945, recall = 0.89, AUC = 0.987.
Clinical Application	Early Diagnosis/Prognosis = recognition of eye diseases using DL. Clinical insights = reliability and enhanced accuracy. TL fine-tunes the model to improve learning efficiency and decision credibility.

The VGG-19 architecture with TL was used in this work to classify eye disease from OCT retinal images [35]. Choroidal neovascularization, drusen, diabetic macular edema, and normal retina are the four retinal diseases that the model categorizes. As shown in Table 13, retinal disorders were accurately detected using the model’s high classification accuracy of 99.17%, sensitivity of 0.99, and specificity of 0.995. In this paper, the researchers used large datasets of OCT images to show that TL improves model performance and becomes an effective tool for the prediction and pre-treatment of retinal disorders.

**Table 13:** Study 13: retinal disease detection using OCT images through a deep learning model.

Category	Description
ML/EEG Features	Dataset = OCT images. Data Analysis = deep convolutional NN with a pre-trained VGG-19 model for TL. EEG Features = classification of the retinal conditions choroidal neo-vascularization, drusen, diabetic macular edema, and normal.
Analysis Method	DL Models = VGG-19 and deep CNN with TL. Task = categorizes OCT retinal images into four retinal conditions. Performance Metrics = classification accuracy = 99.17%, specificity = 0.995, sensitivity = 0.99, AUC, Cohen’s Kappa, and confusion matrix.
Clinical Application	Early Diagnosis/Prognosis= detects retinal diseases with high accuracy, aiding in early diagnosis. Clinical insights = high precision in detecting retinal conditions. TL = Enhances model performance by learning from a large OCT image set.

The authors of [36] focused on improving the pre-trained CNN VGG16 model to categorize RFI to identify diabetic eye disease (DED). As shown in Table 14, the model in these studies achieved a maximum accuracy of 88.3% for multi-class DED and 85.95% for moderate multi-class DED. Techniques for contrast enhancement, optimization, and fine-tuning were used with computational resources that provide an automated technique for the identification of diabetic eye disease. This model can help ophthalmologists work less and improves the precision and effectiveness of clinical diagnostics.

**Table 14:** Study 14: diabetic eye disease detection using RFI.

Category	Description
ML/EEG Features	Dataset = RFI for the diagnosis of diabetic eye disease (DED). Data Analysis = pre-trained CNN-VGG16 model. Features = classification of mild multi-class DED and multi-class DED.
Analysis Method	DL Models = CNN with pre-trained VGG16 model fine-tuned on RFI. Task = classifies RFI into healthy and diseased categories. Performance Metrics = maximum accuracy = 88.3% for multi-class DED, 85.95% for mild multi-class DED.
Clinical Application	Early Detection/Prognosis = automated system for detecting diabetic eye disease, reducing the manual workload of ophthalmologists. Clinical Insights = improved diagnostic accuracy and efficiency in the detection of diabetic eye diseases. Classification Improvement Techniques = fine-tuning, optimization, and contrast enhancement.

Researchers used data of UWF-CFP images to train the DL given in Table 15, employing a multi-layer convolutional neural network (M-CNN) for the classification of DR, SCR, RVO, and healthy eyes [37]. The classification results of the paper showed that AUC remained 90.5% for DR, while it was 91.2% for SCR and 96.7% for SCR, and the HCs’ AUC remained 88.5%. The model’s classification accuracy was 88.4%. The high AUC and accuracy scores from these results show that this DL is very useful for early diagnosis and classification of image dataset EMDs, making the deep CNN model a useful telemedicine algorithm, especially in remote and urban locations where access to ophthalmic care is limited.

**Table 15:** Study 15: Identification of retinal vascular disease (RVD) through ultra-wide-field color fundus imaging.

Category	Description
ML/EEG Features	Data = UWF-CFP images with diabetic retinopathy (DR), sickle-cell retinopathy (SCR), retinal vein occlusions (RVOs), and HCs. Data analysis = multi-layer CNN. Features = classification of DR, SCR, RVO, and healthy eyes.
Analysis Method	DL Models = multi-layer CNN. Classification Task = differentiating between RVO, SCR, DR, and healthy eyes. Performance Metrics = accuracy is 88.4%; DR AUC is 90.5%, accuracy is 85.2%; RVO AUC is 91.2%, accuracy is 88.4%; SCR AUC is 96.7%, accuracy is 93.8%; HCs AUC = 88.5%, accuracy = 86.2%.
Clinical Application	Early prediction potential usage in identifying DR, SCR, and RVOs. Clinical Insights = the high AUC/accuracy DL is effectively classified. This model is a useful tool for telemedicine in areas with limited access to ophthalmic care.

### Cross-Study Synthesis and Methodological Insights

While individual studies report strong performance for specific tasks, a comparative synthesis across the 15 included works reveals several consistent methodological trends and limitations that influence the reported outcomes. Studies employing traditional machine learning (ML) models such as SVM, KNN, Random Forest, and Logistic Regression generally rely on carefully engineered EEG features (e.g., spectral power, coherence, entropy, and synchronization measures). These approaches demonstrate stable performance on small-to-moderate datasets and offer greater interpretability, making them suitable for early-stage clinical investigations and exploratory neurological assessments.

Deep learning (DL) approaches, including CNNs, RNNs, and transformer-inspired architectures, are increasingly adopted in studies with larger datasets or structured representations, such as spectrograms, eye-state segmentation tasks, and multimodal EEG–eye-tracking pipelines. DL models often outperform classical ML methods in capturing complex spatiotemporal dynamics, such as ocular event detection and pheno-conversion prediction. However, their performance gains typically require larger data volumes and substantial computational resources, limiting their immediate clinical scalability.

Feature extraction strategies strongly impact classification performance. Frequency-domain features, particularly power spectral density in the δ, θ, α, and β bands, remain the most widely used EEG descriptors, especially in neurological disorder classification. Connectivity-based measures (e.g., phase lag index and coherence) and entropy-based features are increasingly incorporated to capture network-level and nonlinear dynamics. Multimodal approaches, such as synchronized eye-tracking data or EEG-derived spectrograms, improve robustness by reducing ocular artifacts and enhancing physiological interpretability.

Validation methodologies contribute to notable heterogeneity. Most studies rely on internal cross-validation, with only a few performing external or multi-center validation. This raises concerns regarding generalizability and potential overfitting, particularly for DL-based frameworks trained on small cohorts. Dataset imbalance, limited sample sizes, and variability in recording protocols remain common challenges and can inflate performance metrics in controlled settings.

Additional sources of bias include differences in subject demographics, recording conditions (e.g., eyes open vs. eyes closed), and task-specific designs. Studies using image-based ophthalmic datasets benefit from large-scale availability, whereas EEG-based investigations face acquisition and accessibility constraints. These disparities highlight the need for standardized benchmarks, larger multi-institutional datasets, and transparent reporting of preprocessing and validation procedures.

Collectively, this synthesis underscores that no single modeling paradigm universally outperforms others; rather, performance depends on the interplay between model architecture, feature representation, dataset scale, and validation rigor. Future research should prioritize hybrid ML–DL frameworks, standardized evaluation pipelines, and clinically grounded validation to ensure reliable translation of AI-driven EEG analysis into real-world neurological and oculomotor disorder assessment.

The relative effectiveness of models and features depends on the neural and oculomotor characteristics of each task. For instance, CNNs paired with spectral features are particularly effective for disorders like RBD, where disease-specific alterations manifest as stable spatial or frequency patterns. In contrast, tasks such as MDD classification or eye-state detection involve subtler temporal dynamics, making RNNs or hybrid DL architectures more suitable. This task–feature–model alignment emphasizes that performance is context-dependent rather than universally superior for any single approach.

Emerging best practices include standardized EEG preprocessing workflows, robust handling of eye movement and ocular artifacts (e.g., ICA, regression-based correction, EEG–eye-tracking integration), and strategies to enhance model interpretability, such as attention mechanisms, feature visualization, and explainable AI approaches. Adoption of these practices is expected to improve the reproducibility and clinical relevance of EEG-based ML studies.

## 4. Conclusions

This systematic review has examined recent advances in AI-driven analysis of electroencephalography (EEG) signals for the assessment of neurological and oculomotor disorders. Across the 15 included studies, EEG-based machine learning and deep learning models were applied to a broad range of clinical contexts, demonstrating the versatility of EEG as a non-invasive biomarker for disease classification, prognosis, and monitoring. The findings collectively indicate a clear methodological evolution from traditional machine learning approaches toward more sophisticated deep learning architectures capable of capturing complex spatiotemporal neural patterns.

Several studies highlighted the effectiveness of EEG-based AI models in identifying disease-specific neural signatures, supporting early diagnosis and subtype differentiation in neurological conditions. In parallel, emerging work on ocular and oculomotor-related applications illustrates the potential of EEG-driven approaches to complement conventional imaging and behavioral assessments. Despite promising performance across multiple evaluation metrics, most studies relied on relatively small or homogeneous datasets, limiting their generalizability and clinical readiness. While these studies show potential for early diagnosis and clinical application, the limited dataset sizes and lack of cross-site validation suggest that these findings should be interpreted cautiously, and further validation in larger, multi-center cohorts is required before clinical translation.

Common challenges identified in the literature include inconsistent validation protocols, limited external testing, and variability in feature extraction and preprocessing strategies. These issues underscore the need for standardized methodological frameworks, larger multi-center datasets, and rigorous validation to ensure robustness and reproducibility. Future research should prioritize clinically interpretable models, cross-population evaluation, and integration with multimodal data sources to enhance real-world applicability.

In conclusion, AI-driven EEG analysis represents a rapidly evolving and promising avenue for advancing neurological and oculomotor disorder assessment. Continued methodological refinement and collaborative data-sharing efforts will be essential for realizing its full potential in clinical diagnostics, personalized treatment planning, and long-term patient monitoring.

## Data Availability

Not applicable.
